# Experimental infection of rabbits with bovine viral diarrhoea virus by a natural route of exposure

**DOI:** 10.1186/1297-9716-45-34

**Published:** 2014-04-02

**Authors:** Claudia Bachofen, Dawn M Grant, Kim Willoughby, Ruth N Zadoks, Mark P Dagleish, George C Russell

**Affiliations:** 1 Moredun Research Institute, Pentlands Science Park, Penicuik EH26 0PZ, UK; 2 Present address: Institute of Virology, Vetsuisse faculty, University of Zurich, Winterthurerstr 266a, CH-8057 Zurich, Switzerland

## Abstract

Bovine viral diarrhoea virus (BVDV) is an important pathogen of cattle that can naturally infect a wide range of even-toed ungulates. Non-bovine hosts may represent reservoirs for the virus that have the potential to hamper BVDV eradication programs usually focused on cattle. Rabbits are very abundant in countries such as the United Kingdom or Australia and are often living on or near livestock pastures. Earlier reports indicated that rabbits can propagate BVDV upon intravenous exposure and that natural infection of rabbits with BVDV may occur but experimental proof of infection of rabbits by a natural route is lacking. Therefore, New Zealand White rabbits were exposed to a Scottish BVDV field strain intravenously, oro-nasally and by contaminating their hay with virus. None of the animals showed any clinical signs. However, the lymphoid organs from animals sacrificed at day five after exposure showed histological changes typical of transient infection with pestivirus. Most organ samples and some buffy coat samples were virus positive at day five but saliva samples remained negative. Development of antibodies was observed in all intravenously challenged animals, in all of the nebulised group and in four of six animals exposed to contaminated hay. To our knowledge this is the first report of BVDV propagation in a species other than ruminants or pigs after exposure to the virus by a natural route. However, to assess the role of rabbits as a potential reservoir for BVDV it remains to be determined whether persistent infection caused by intra-uterine infection is possible and whether BVDV is circulating in wild rabbit populations.

## Introduction

Bovine viral diarrhoea virus (BVDV) type 1 and type 2, together with classical swine fever virus (CSFV) and Border disease virus (BDV) are the main species in the genus *Pestivirus* within the family *Flaviviridae*. BVDV, CSFV and BDV are pathogens of even-toed ungulates, infecting primarily cattle, pigs and sheep respectively [1]. A unique characteristic of pestiviruses, particularly relevant in the case of BVDV, is the generation of persistently infected (PI) and immunotolerant offspring after transient infection of the dam during a critical window of gestation [2]. PI animals shed virus throughout their lives without producing an immune response and are the most important source of BVDV infection for immunologically naïve cattle [3]. BVDV PI animals may show no clinical signs as calves, however, they often have reduced growth and productivity and their life-expectancy is significantly reduced. Herds with BVDV generally have reduced reproductive performance and a higher rate of diseases such as scour and pneumonia [4]. Because of the economic losses due to BVDV infection, many European countries have undertaken eradication programmes. Pioneered by Scandinavian countries, national compulsory eradication programmes are ongoing in Austria, Switzerland, Germany, Ireland and Scotland and are based on detection and removal of PI animals with or without vaccination of uninfected animals in the herd [5]. In several other countries, regional and voluntary programmes exist. While eradication is usually very efficient in the first years after implementation, leading to a rapid decrease in the prevalence of PI animals, the final stage is notoriously long and characterised by re-infections of previously BVDV-free herds [6]. The reasons for this are multiple: decreasing seroprevalence in cattle herds facilitates the spread of new infections due to issues such as non-compliance with movement restrictions on potentially infected animals or false-negative results in tested animals. Another source of re-infection may be virus reservoirs in non-bovine hosts. BVDV can cross the species barrier relatively easily, particularly into sheep, where it causes a disease clinically indistinguishable from that caused by border disease virus [7]. Antibodies against BVDV have been detected in a wide range of wild and domesticated ruminant and porcine species [8-11] and persistent infection has been demonstrated in sheep, goats, pigs, alpaca, white-tailed deer, eland, mouse deer, and American mountain goats [11-18]. In the early years of BVDV research, a wide range of non-artiodactyls such as horses, cats, dogs, several small laboratory animal species (guinea pig, mouse, rabbit) and embryonated chicken eggs were also inoculated with the virus in order to determine the host range [19]. The only non-artiodactyl animal in which virus could be propagated upon intravenous inoculation was the rabbit. Baker et al. [19] reported that calves inoculated with spleen homogenate from rabbits that had been infected with BVDV five days earlier showed clinical signs typical of transient BVDV infection. Furthermore, BVDV could be serially passaged, both within rabbits and between rabbits and cattle, using lymphoid cell suspensions [20]. More recently, a serological survey in Germany showed that 40% of sera sampled from 100 wild rabbits exhibited low neutralising antibody titres against BVDV [21]. However, only a third of the positive results could be confirmed by ELISA and no virus could be isolated from any rabbit.

Thus, there are indications that rabbits could be hosts for natural BVDV infection, but clear experimental or epidemiological evidence is missing. Since rabbits are very abundant in countries such as the United Kingdom and Ireland, often living on or near livestock pastures, a BVDV reservoir in rabbits could have significant consequences for BVDV eradication campaigns in these countries, especially towards the end of an eradication scheme. Therefore, in order to investigate the role of rabbits as potential reservoir hosts of BVDV, we exposed rabbits to BVDV by different routes; intravenously, oro-nasally and by contaminating their bedding with virus. Our results indicate that rabbits can indeed be infected by BVDV, not just intravenously but also by more natural routes of infection.

## Materials and methods

### Animals and experimental design

**Figure 1:**
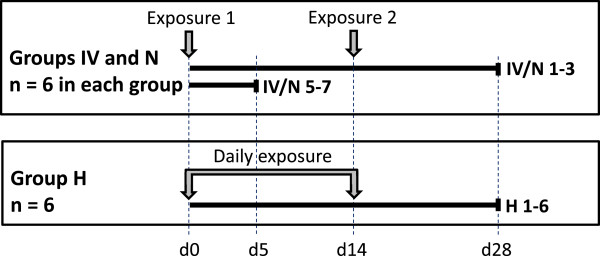
**Overview of the experimental design.** Rabbits were exposed to bovine viral diarrhoea virus (BVDV) intravenously (IV), by nebuliser (N) or by contaminated hay (H). Animals 4 and 8 from groups IV and N were mock exposed (negative controls). The two sentinel rabbits of group H that received normal hay are not depicted in the figure. Animals IV/N5-8 were sacrificed at day five after exposure, all other animals at day 28 after initial exposure. Rabbits of groups IV and N were re-exposed after 14 days, rabbits of group H were exposed daily during the initial 14 days.

Twenty-four 12 week old male New Zealand White rabbits were purchased from a certified breeder. The animal experiments were carried out with the approval of the Experiments and Ethics Committee of the Moredun Research Institute and complied fully with the Home Office of Great Britain and Northern Ireland’s implementation of “Animals (Scientific Procedures) Act 1986”. The rabbits were acclimatised for one week prior to being assigned randomly into three groups of eight animals that were housed in individual boxes, with each group in a separate room. The three groups were exposed to BVDV by three different routes: intravenously via ear vein (IV group), oro-nasally by a nebulizer device using a soft anaesthetic mask (N group) and by contaminating the hay provided in each box (H group). An overview of the experimental design is given in Figure 1. In the IV and N groups, six animals were inoculated or nebulised with 1 mL of virus (10^6^ TCID50) whilst two animals in each group were mock infected with 1 mL of tissue culture medium (Iscove’s modified Dulbecco’s medium (IMDM), Sigma-Aldrich Company Ltd. Dorset, UK). The inoculum of 10^6^ TCID50 was chosen as this amount of virus was shown previously to induce transient infection in cattle [22]. Inoculation/nebulisation was repeated after 14 days. In group H, six animals were given hay that was contaminated with BVDV (1 mL of virus; 10^6^ TCID50) using a syringe-fitted atomisation device (Intavent Direct, Old Amersham, UK). The hay was replenished with freshly BVDV contaminated hay daily for 14 days and two animals which received normal hay were kept in the same room as sentinels for further aerosol spread of virus. The body temperature of each animal was monitored daily by a subcutaneous microchip placed in the neck region (idENTICHIP; Animalcare, York, UK). Blood samples were taken from the ear vein into EDTA anticoagulant at day 0, prior to exposure, and on days 3, 5, 8, 14, 22 and 28 after first exposure. At the same time points, oral swab samples were taken (FLOQSwab MiniTip, Copan, Brescia, Italy) and immediately transferred to centrifuge tubes containing 1 mL of IMDM. At day five, when active viral replication might be expected in BVDV-infected animals, one mock-exposed and three virus-exposed animals from each of groups IV and N were euthanized for collection of organ samples and cardiac blood. Samples of spleen, liver, kidney, ileum (sacculus rotundus), lung, heart, whole brain and appendix for histopathology and immunohistochemistry were fixed in buffered formal saline and Zinc salts fixative [23], processed to paraffin blocks and stored at 4 °C. For detection of BVDV viral RNA and virus isolation, samples of ileum (sacculus rotundus), appendix and spleen were frozen under aseptic conditions and stored at -80 °C. Because of the unknown timing of the onset of infection in group H, no animals were sacrificed at day five. All remaining animals were euthanized at the end of the study (day 28). As it was unlikely to detect any virus in the organs at this late time point after exposure, only cardiac blood samples (EDTA and coagulated) were taken from the animals euthanized at day 28.

### Virus

The BVDV isolate (MRI103) used for the experimental exposures was isolated from the serum of a Scottish PI bovid which was free of maternal antibodies, and passaged six times on bovine turbinate (BT) cells. After three passages, the virus was titrated on BT cells and a multiplicity of infection (MOI) of 0.01 was used for the following passages as previously described [21]. Medium from the sixth passage, containing BVDV at a titre of 10^6^ TCID50/mL, was clarified by centrifugation at 4000 × *g* for 30 min and stored in aliquots at -80 °C before use. All cells, tissue culture medium (IMDM) and foetal bovine serum (FBS) used were tested free of pestivirus and antibodies against pestivirus. The 5′UTR and N^pro^ coding region of the isolate were sequenced for phylogenetic typing as previously described [24] and MRI103 was determined to be a BVDV-1a virus.

### RNA isolation and BVDV Real-time RT-PCR

RNA isolation from EDTA blood and swab samples was performed using a viral RNA mini kit (QIAGEN Ltd., Manchester, UK) according to the manufacturer’s instructions. For tissue samples, homogenisation of about 30 mg of frozen tissue by ceramic beads in RLT buffer (QIAGEN) using the Precellys 24 tissue homogenizer was followed by RNA isolation using the RNeasy mini kit (QIAGEN). Buffy coats from blood samples of animals sacrificed at day five were isolated using a commercial red cell lysis buffer (Promega UK Ltd, Southampton, UK). Subsequent RNA isolation was performed using QIAShredder columns and the RNeasy mini kit (QIAGEN). For detection of viral RNA an established real time RT-PCR was used [25]. BVDV-1 specific and beta-actin RNA were detected in separate assays on an ABI 7500 sequence detection system (Applied Biosystems-Life Technology Ltd., Paisley, UK). Positive RNA samples (Ct < 40) were retested to confirm the result.

### Virus isolation

Approximately 30 mg of each ileum sample taken at day five after exposure were homogenised by ceramic beads in 800 μL of IMDM in a Precellys 24 tissue homogenizer (Stretton Scientific Ltd., Derbyshire, UK). Cell debris was pelleted by centrifugation at 4000 × *g* for 30 min at 4 °C, the supernatant mixed with an equal volume of IMDM and inoculated onto BT cells pre-seeded in 25 cm^2^ tissue culture flasks. After incubation for one hour at 37 °C, the inoculum was removed and replaced by fresh IMDM containing 2% FBS. Following a four day incubation, the cells were freeze-thawed and the suspension clarified by centrifugation at 4000 × *g* at 4 °C for 30 min. The supernatant was diluted 1:2, 1:10, 1:100 and 1:1000 in IMDM and transferred to a 96-well microtitre plate containing BT cells (two wells per dilution). After four days of incubation, BVDV viral protein was visualised in infected cells by immunoperoxidase staining as described previously [26].

### ELISA for detection of BVDV antibodies

A biphasic, indirect antibody capture ELISA was used to detect BVDV antibodies in plasma samples. The test was used essentially as described previously [27] except that the horseradish peroxidase (HRP) labelled anti-bovine detection antibody was replaced with a polyclonal goat anti-rabbit HRP antibody (#P0448, Dako UK Ltd., Cambridgeshire, UK). Briefly, alternate columns of a 96-well ELISA plate (high binding, Greiner Bio-One Ltd., Gloustershire, UK) were coated with antigen from Igepal treated BVDV (isolate C24V) infected cells or with an equivalent antigen preparation from uninfected cells. Prior to usage, plates were blocked for 45 min at room temperature with a solution of 4% milk powder in PBS containing 0.05% Tween20 (PBST). The rabbit plasma samples were diluted 1:10 in PBST containing 2% milk powder and added in quadruplicates to the plate. After incubation (1 h) and washing, the HRP labelled anti-rabbit detection antibody (diluted at 1:1000) was added. Following a further 1 hour incubation and a wash step, bound antibody was visualised by adding Tetramethylbenzidine (TMB) substrate (SureBlue, KPL Inc., Gaithersburg, USA). The reaction was stopped after five minutes by addition of 0.18 M sulphuric acid and absorbance at 450 nm was measured in an ELISA plate reader (Dynex MRX^e^, Dynex Technologies Ltd., West Sussex, UK). Since no certified positive or negative rabbit control sera were available, aliquots of pooled positive terminal plasma from the three BVDV exposed rabbits of the intravenous group (IV 1, 2 and 3) that were positive in a serum neutralisation test (SNT) were used to calculate the relative optical density (OD) values (expressed as a sample-to-positive ratio (S/P value)). Pooled pre-exposure plasma from the mock-infected rabbits (IV4 and N4) was used as the negative control. The following formula was used to calculate S/P values, where the corrected OD is the mean OD of positive antigen wells minus the mean OD of the negative antigen wells inoculated with the same sample:

$$\begin{array}{l} S/P=\left(\text{corrected}\;\text{OD}\;\text{of}\;\text{sample}‒\text{corrected}\;\text{OD}\;\text{of}\;\text{negative}\;\text{control}\right)/\\ \;\left(\text{corrected}\;\text{OD}\;\text{positive}\;\text{control}‒\text{corrected}\;\text{OD}\;\text{of}\;\text{negative}\;\text{control}\right) \end{array}$$

### Serum neutralisation test (SNT)

SNT of terminal sera was performed as previously described for cattle sera [24]. The NADL BVDV strain was used to determine neutralising antibody titres on BT cells. As NADL is a cytopathic virus, a cytopathic effect (CPE) could be determined microscopically in infected cells. However, to exclude non-specific CPE, the cells were additionally stained for BVDV antigen by immunoperoxidase staining as described for virus isolation.

### Histopathology and immunohistochemistry (IHC)

Tissue samples for histopathology (brain (coronal slices through the anterior pole of the cerebrum, corpus striatum, thalamus, occipital lobes and mid-brain, cerebellar peduncles and three levels of the medulla along with a sagittal section through the cerebellar vermis), lung, heart, liver, spleen, ileum (sacculus rotundus), appendix and kidney) from one negative control and three BVDV challenged animals from groups IV and N killed at day five were processed routinely (dehydrated through graded alcohols, embedded in paraffin wax, sectioned (5 μm), mounted on glass microscope slides and stained with haematoxylin and eosin (HE)) for examination by light microscopy. Further sections were subjected to IHC for BVDV (a single brain section (through the occipital lobes and mid-brain) and all other viscera). Briefly, all sections were mounted on Superfrost™ slides (Menzel-Gläser, Braunschweig, Germany), dewaxed in xylene and rehydrated through graded alcohols to 95% alcohol prior to quenching endogenous tissue peroxidase activity with 3% hydrogen peroxide in methanol (v/v) for 20 min. Subsequent to this, slides were washed in water for 5 minutes prior to antigen retrieval using Proteinase K (Dako) (20 μg/mL in Tris–HCl pH 7.6 for 5 min at room temperature). Non-specific antigen binding was blocked by incubation with 25% normal goat serum (Vector Laboratories, Peterborough, UK), diluted in tris-buffered saline pH 7.6 (TBS), for 30 min at room temperature (approx. 18–22 °C) prior to addition of the primary antibody; monoclonal mouse anti-pestivirus glycoprotein-48 (clone 15C5, [28]) diluted 1/40 000 in TBS and incubated overnight at 4 °C. Slides were washed in TBS and primary antibodies visualised using the EnVision™ System-HRP (DAB) (code K4007, Dako) as per manufacturer’s instructions prior to being washed in water, counterstained with haematoxylin Z (Cellpath plc., Powys, UK), blued-up with Scot’s tap water substitute, dehydrated, cleared and mounted. Negative control slides were prepared by substituting species and isotype matched IgG at the same dilution as the primary antibody.

## Results

### Detection of virus in rabbits exposed to BVDV

Following exposure to BVDV, none of the animals showed any clinical signs, or significant changes in body temperature, at any point during the experiment. The oral swab samples taken at days 0, 3, 5, 8, 14, 22 and 28 were BVDV RNA negative by real time RT-PCR but positive for beta-actin RNA. Thus, we could not detect any evidence of viral shedding by the oral route in the animals. Samples of EDTA-anticoagulated whole blood taken at the same time points were also BVDV negative and beta-actin positive. However, buffy coats isolated from blood samples from the rabbits sacrificed at day five of infection were BVDV positive in two of the three BVDV exposed animals from the IV group (Table 1). In these animals Ct values ranged from 38.5 to 40.0 (data not shown). Because of its known involvement in BVDV infection in cattle, the organs of the gut associated lymphoid tissue (GALT) were tested for the presence of the virus. In rabbits, the main GALT is in the appendix and the sacculus rotundus, a sac-like expansion of the ileum at the ileocaecal junction. In addition, spleen was examined for BVDV as previous studies have shown that BVDV could be transmitted from rabbits to calves by spleen homogenate [19]. All of the organs examined from each of the three BVDV challenged rabbits in the IV group tested at day 5 were positive by real time RT-PCR (Table 1) while the organs of the mock challenged rabbit (IV8) were all negative. The lowest Ct values were observed in ileum (24.3 - 30.5) and appendix (25.6 - 33.6) while less viral RNA was detected in spleen (35.6 - 36.7). In the nebulised group, one of three sacrificed animals was BVDV positive in all three organs (N5), one in appendix and spleen (N6) and one in spleen only (N7). The Ct values ranged from 33.0 (ileum) to 38.7 (spleen). None of the organs of the mock exposed rabbit in this group (N8) were BVDV positive. Since the onset of infection in the hay group was not predictable, none of these rabbits were euthanized at day five. Therefore, we have no indication about the presence of BVDV in the organs of this group at this time point. To determine whether the positive RT-PCR results were indicative of infectious virus or only of the presence of viral RNA, we inoculated BT cells with ileum (sacculus rotundus) homogenate from IV group animals. Infectious virus was detectable in organ samples from the BVDV-challenged animals whilst the ileum of the mock challenged rabbit was virus negative (Table 1).

**Table 1:** Bovine viral diarrhoea virus (BVDV) detection and pathology in rabbits at day 5 after challenge by intravenous route (IV) or nebulizer (N)

	**BVDV RT-PCR**^**a**^				**Virus isolation**	**GALT depletion**^**c**^
**Animal**	**Buffy coat**	**Ileum**	**Appendix**	**Spleen**	**Ileum**	
IV5	neg	+	+	+	+	moderate-severe
IV6	+	+	+	+	+	mild-moderate
IV7	+	+	+	+	+	moderate-severe
IV8^b^	neg	neg	neg	neg	neg	none
N5	neg	+	+	+	not done	indeterminate^c^
N6	neg	neg	+	+	not done	mild-moderate
N7	neg	neg	neg	+	not done	mild-moderate
N8^b^	neg	neg	neg	neg	not done	indeterminate^c^

^a^RT-PCR for viral RNA detection was performed as described in the materials and methods. In each case, 1 μL of total RNA was used in assays specific for BVDV type 1 and for genomic β-actin. Assays with Ct for BVDV and for actin less than 40 were considered positive (+), while assays with Ct for BVDV greater than 40.1 and Ct for actin less than 40 were considered negative (neg).

^b^negative control animals.

^c^Gut associated lymphoid tissue (GALT) depletion was assessed by histopathology as described in the materials and methods section. In group N, GALT depletion could not be determined clearly in two animals due to autolysis.

### Serological analysis of rabbit immune response to BVDV infection

**Figure 2:**
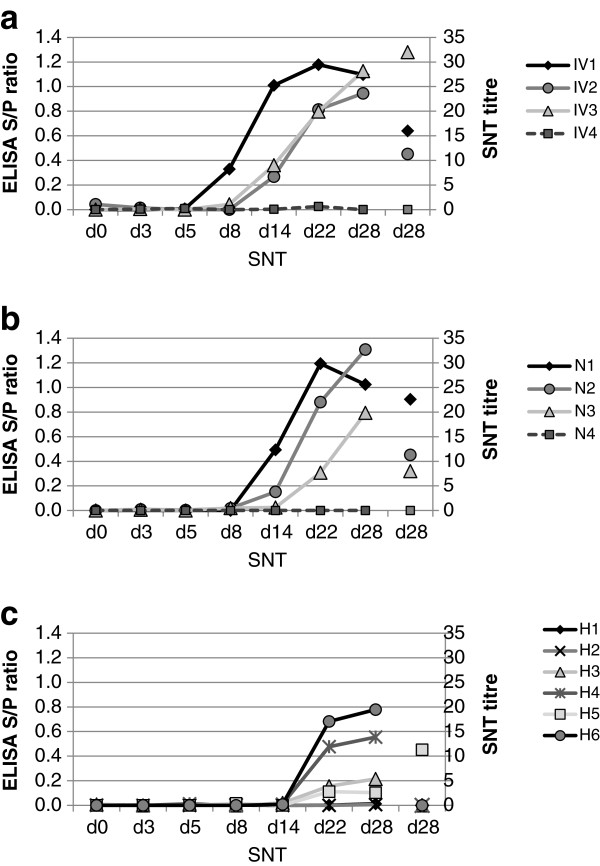
**Virus-specific antibody titres in rabbits exposed to bovine viral diarrhoea virus (BVDV).** A modified in-house antibody capture ELISA was used to measure antibodies against BVDV in plasma of rabbits from groups challenged: **(a)** intravenously (IV); **(b)** by nebuliser (N); and **(c)** through exposure to contaminated hay (H) (Figure 1). Samples for analysis were taken prior to exposure (d0) and at different time points after exposure (d3, 5, 8, 14, 22, 28). IV/N4 (dashed lines) were mock exposed rabbits. Neutralising antibody titres of terminal sera were determined by SNT (d28 SNT).

For detection of a BVDV-specific antibody response in the exposed rabbits, plasma samples from all rabbits at days 0, 3, 5 and those remaining at days 8, 14, 22 and 28 were tested in a modified in-house BVDV antibody capture ELISA. As shown in Figure 2, the S/P ratio of all BVDV exposed animals of group IV (IV1-3) increased significantly after day five while the mock exposed rabbit (IV4) remained negative. The response was very similar in the nebulised rabbits, except that the onset of the antibody response seemed to be slightly delayed compared to the IV group. In group H, a virus-specific antibody response became detectable only after day 14. In this group, four of the six exposed animals showed an increase in the S/P ratio. Blood samples from the two sentinel rabbits, kept in the same room as group H, were only taken at day 28. These were both BVDV antibody negative in the ELISA (data not shown). To determine the virus neutralising capacity of the antibodies, an SNT was performed on serum samples taken at day 28 (Figure 2). Due to the small volumes of blood collected *ante-mortem*, insufficient serum was available from the earlier sampling time points. The BVDV exposed rabbits showed neutralising antibody titres between 11 and 32 (IV group) and between 8 and 23 (N group), whilst a single animal in group H (H5) showed a virus-neutralising titre of 11. No neutralising antibodies (titres < 4) were detected in the mock exposed rabbits (IV4 and N4).

### Pathological analysis of BVDV infected rabbits

**Figure 3:**
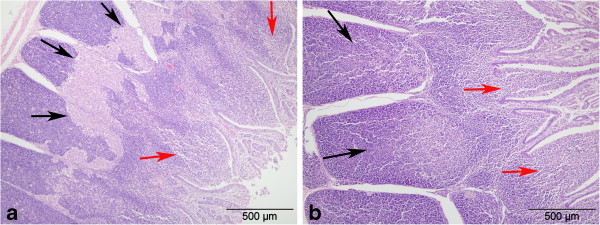
**Haematoxylin and eosin stained sections from infected and control rabbit small intestine.** Histological appearance of ileum in rabbits challenged by the intra-venous route with **(a)** bovine viral diarrhoea virus (BVDV) or **(b)** tissue culture medium (negative control). Intestinal lumen to the right and serosal surface to the left in both photomicrographs. a) note multiple areas of severe lymphoid cell depletion in the Peyer’s patches that had coalesced (black arrows) and were infiltrated by macrophages. Mild lymphoid cell depletion was present in the sub-epithelial domes (red arrows) also. b) note well-populated lymphoid tissue (black arrows) and the sub-epithelial domes (red arrows).

Histopathological analysis of all organs taken at day five revealed no pathological changes in brain, lung, heart, liver and kidneys. In contrast, variably mild to severe lymphoid cell depletion was observed in the GALT of ileum and appendix in all the BVDV-exposed but not in the mock-exposed rabbits of the IV group (Figure 3; Table 1). Unfortunately, the intestinal tissues of two rabbits of the group N had become slightly autolytic before fixation and therefore a clear differentiation of lymphoid depletion and autolysis was not possible. All tissues from all animals killed at day 5 were devoid of labelling for BVDV antigens by immunohistochemistry. Brain samples from known BVDV positive calves (PI used as positive controls) showed extensive cell associated labelling.

## Discussion

BVDV is typically a pathogen of cattle but is not restricted to this host. However, BVDV was generally thought not to infect non-artiodactyls. Experimental intra-venous exposure of a range of species suggested that rabbits could propagate the virus [19,20]. In addition, serological studies have suggested that natural infection of rabbits by BVDV may occur [21]. However, experimental exposure by a natural route has never been tested and the role of the rabbit as a natural host for BVDV infection remained controversial. Nevertheless, the abundance of rabbits on livestock pastures in countries and nations that are trying to eradicate BVDV, such as the Republic of Ireland and Scotland, make an improved understanding of BVDV infection of rabbits advisable.

Therefore, rabbits were challenged with BVDV by different routes and the development of viraemia and virus-specific antibody responses were monitored (Figure 1). Intravenous inoculation of virus was used as a positive control because this had been reported previously as a successful route of BVDV infection of rabbits [20], whereas oro-nasal administration of virus by nebulisation represented a more natural route of virus entry, which allowed some degree of control over the dose of virus administered to each animal. Following previous studies which indicated that repeated pestivirus exposure facilitated interspecies transmissions [29-31], we re-challenged the IV and N group animals after 14 days. A third group of animals was exposed to BVDV by a potentially natural cattle-to-rabbit route, virus-contaminated hay, which was replenished daily for the first two weeks of the experiment. Since the rabbits were fed on dry pellets, the hay was primarily used as a source of long fibre and as bedding material.

The virus used for all exposures was not an established, tissue culture adapted, laboratory strain but a Scottish field isolate that was minimally passaged in order to maintain quasispecies diversity of the infecting virus. In addition to providing better representation of the natural situation of infection from a PI animal, using a virus with a degree of quasispecies diversity may be important for interspecies transmission as indicated by in vitro and in vivo experiments [32,33].

None of the exposed animals showed any clinical signs or elevated body temperature upon infection, in accordance with the previous description [19]. This seems remarkable in view of the viraemia and depleted GALT observed in some animals. Viral RNA was not detected in whole blood samples but could be detected in several buffy coat samples isolated from animals in the IV and N groups at day five (Table 1). This suggests that BVDV load during viraemia is low and primarily cell-associated. This is comparable to the situation in transiently infected cattle where viraemia occurs at a similar time after infection to that which we observed for these rabbits [22] and is usually short lived. Notably, viraemia in cattle is not easily detectable in all cases and virus shedding is very limited [34]. It is therefore not surprising that BVDV viral RNA was not detected in the oral swab samples from the rabbits. In contrast, BVDV RNA was detected in several organ samples at day five (Table 1) and in the spleen samples of all BVDV exposed rabbits in the IV and N groups. As the viraemia appears to be primarily associated with leukocytes, frequent detection in the spleen is not surprising and explains why intravenous virus transmission using spleen homogenate was successfully used in previous experiments [19,20]. It is worth noting that the lowest Ct values (and thus the highest viral load) was detected in the sacculus rotundus part of the ileum (data not shown), which is the rabbit equivalent to the Peyer’s patches in cattle. This tissue was therefore used for virus isolation and, in all cases analysed, infectious BVDV was detected (Table 1). In PI cattle, the virus is typically found in the Peyer’s patches, which can suffer severe lymphoid depletion following the onset of mucosal disease [35]. Mild to moderate depletion of the Peyer’s patches was also reported in transient infection of cattle with BVDV [36]. Similarly, mild to severe depletion of the GALT in ileum and appendix was observed in all BVDV exposed rabbits where tissue quality was adequate (Figure 3; Table 1).

Following the detection of viraemia at day five, an antibody response was detectable in all BVDV exposed rabbits of IV and N groups by day 14 but not in the mock exposed animals (Figure 2). Since there is no validated BVDV ELISA for rabbit sera available, definition of seroconversion was difficult due to the lack of data to allow cut-off values to be established. However, the modified ELISA used here was a biphasic assay, so that false positive reactions due to non-specific antibody binding to components of the coating antigen or the reaction plate can be largely ruled out. Furthermore, no BVDV-specific reactivity was detected in any pre-exposure samples and no increase in S/P values was observed in plasma from mock-exposed rabbits. Even though we cannot exactly determine the time point of seroconversion in the rabbits, the onset of the antibody increase was observed between days five and 14. The time course of the antibody response measurable by ELISA therefore appears similar to that observed in cattle, where seroconversion is reported to occur between two to three weeks after infection [37]. While the in-house ELISA used here is thought to detect mainly antibodies against the conserved non-structural NS3 (p80) protein, produced only during active virus replication [38], we cannot exclude a contribution from antibodies against structural proteins. However, retesting of plasma samples from day 28 in a modified commercial blocking ELISA specific for the detection of anti-NS3 antibodies (BVDV/MD/BDV p80 Protein Antibody Test Kit, Idexx) confirmed the results of the in-house ELISA (data not shown) and showed that the antibody reaction measured was not simply due to a hyperimmune reaction against virus particles. In contrast to ELISA, SNT detects mainly antibodies against the structural E2 envelope protein [39]. Interestingly, while the ELISA results from rabbits seemed to be similar to the time course and degree of antibody response in transiently infected cattle, the SNT titres after 4 weeks were low (Figure 2). In cattle sera from field cases, titres are reported to be about ten times higher [24]. However, levelling off of the SNT titre in cattle is only reached 10–12 weeks after infection [37]. Thus, the neutralising titres in rabbits might have reached higher titres if the animals had been maintained for a longer time. The virus strain used for the SNT was of the same BVDV-1a subgroup as the virus used for challenging the rabbits, antigenic differences should therefore not be the reason for the low titres. The E2 protein is known to be the main determinant of host species selection in pestiviruses [40] and the E2 coding region is known to be highly variable within and between BVDV isolates [33,41]. Sequencing of virus recovered from the experimentally infected rabbits would show if changes in the E2 coding region upon interspecies transmission may contribute to the low neutralising titres. However, the virus neutralising antibody titres described previously in wild rabbits [21] were very similar to those observed here in the experimentally infected rabbits. It is unlikely that these low titres would confer protection against re-infection, particularly with a different BVDV isolate [42]. However, in addition to neutralising antibodies, cellular immunity is known to be important for protection against re-infection in cattle [37]. Further experiments are necessary to determine the quality and duration of immune protection in rabbits.

In summary, our results indicate that rabbits are susceptible to infection by BVDV and that infection does not cause clinically apparent disease. The evidence of viraemia and the detection of anti-NS3 antibodies strongly suggest the virus can be propagated in rabbits. Importantly, evidence for virus propagation was found both after intravenous infection and also when the animals were exposed to the virus oro-nasally. Although infection was less successful in the rabbits exposed to virus-contaminated hay, even this indirect route of transmission led to an anti-BVDV immune response, as measured by ELISA, in four out of six animals (Figure 2). While some aspects of infection seem to be similar to infection in cattle, such as the time course of antibody development and the targeting of GALT organs, others are clearly different, such as the poor production of neutralising antibodies and the lack of clinical signs. To our knowledge this is the first report of BVDV infection by a natural route of animals other than even-toed ungulates and it highlights the flexibility of BVDV with regard to host range. However, in order to determine the role of rabbits as a potential reservoir for BVDV, further experiments are necessary to show whether persistently infected rabbits can be produced to generate cattle-independent chains of infection. Furthermore, BVDV ELISA analyses of sera from wild rabbits from regions with different cattle densities may provide additional information on the epidemiology of BVDV in rabbits in the field.

## Competing interests

The authors declare that they have no competing interests.

## Authors’ contributions

Experimental design and planning: GCR, CB, DMG, RNZ, KW; animal experiments: CB, DMG; virological and serological laboratory work: CB, KW; histopathology: MPD; data processing and drafting of manuscript: CB, GCR; All authors read, edited and approved the manuscript.
